# Supplementary material for: Implications of dominance hierarchy on hummingbird-plant interactions in a temperate forest in Northwestern Mexico
Source: PeerJ. 2023 Oct 17;11:e16245. doi: 10.7717/peerj.16245 (PMC10588686; doi:10.7717/peerj.16245)
Supplement: Supplemental Information 1 — Values that exceed 0.6 are a strong indicator of dominance among interactions. Axis values represent individual dominance rank, with 1 indicating the top-ranked hummingbird species. [file peerj-11-16245-s001.pdf]

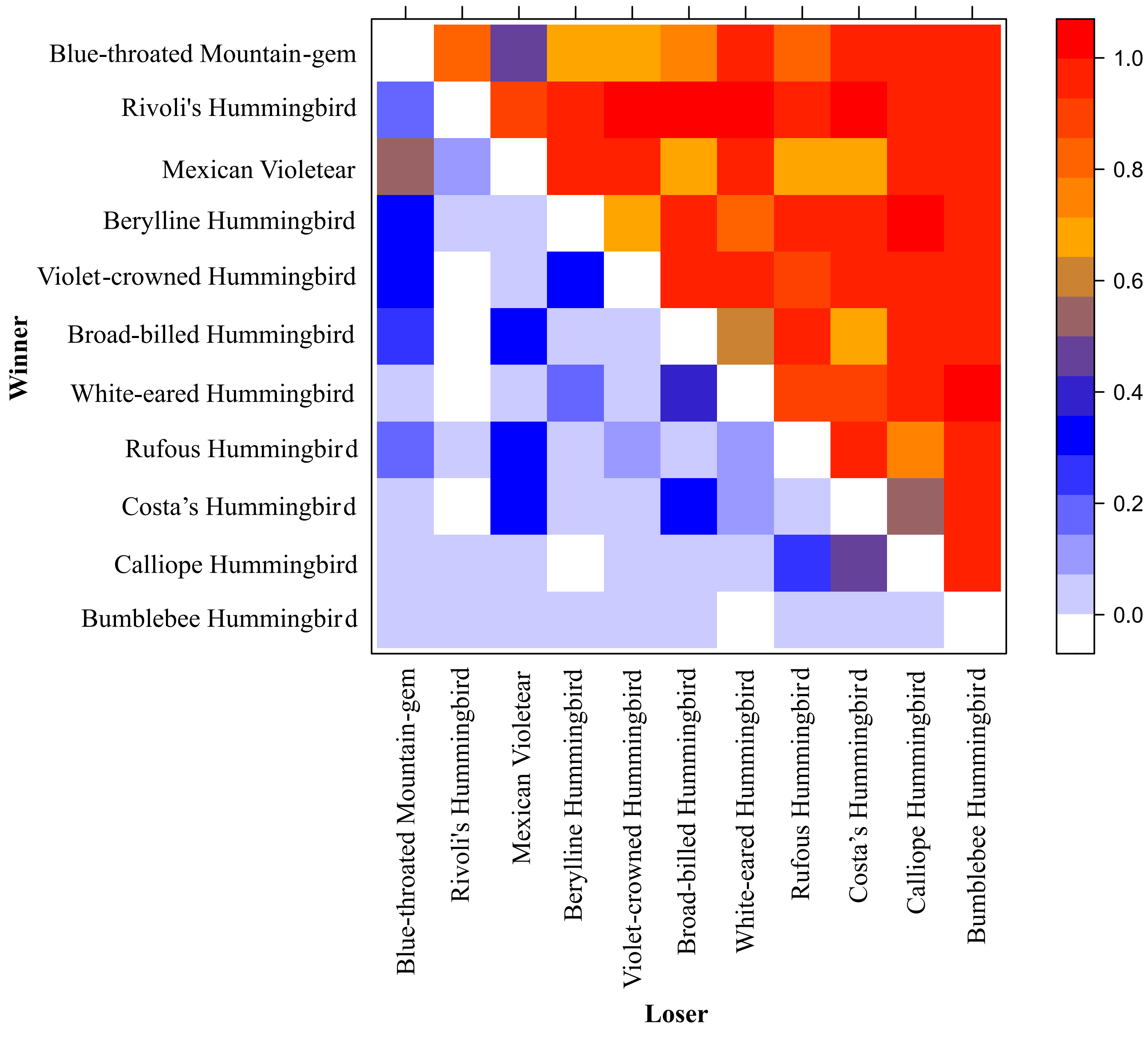
Winner
Loser
Blue-throated Mountain-gem
Rivoli's Hummingbird
Mexican Violetear
Berylline Hummingbird
Violet-crowned Hummingbird
Broad-billed Hummingbird
White-eared Hummingbird
Rufous Hummingbird
Costa's Hummingbird
Calliope Hummingbird
Bumblebee Hummingbird
0.0
0.2
0.4
0.6
0.8
1.0
