# Supplementary material for: Implications of dominance hierarchy on hummingbird-plant interactions in a temperate forest in Northwestern Mexico
Source: PeerJ. 2023 Oct 17;11:e16245. doi: 10.7717/peerj.16245 (PMC10588686; doi:10.7717/peerj.16245)
Supplement: Supplemental Information 3 [file peerj-11-16245-s003.docx]

Table S1. Hummingbird-plant visitation matrix, during 338 h of focal observations.

|  | Ruby-throated | White-eared | Costa’s | Broad-billed | Mexican | Rivoli's | Blue-throated | Violet-crowned | Berylline | Calliope | Bumblebee | Broad-tailed | Rofous |
| --- | --- | --- | --- | --- | --- | --- | --- | --- | --- | --- | --- | --- | --- |
| *A.inaequidens* | 0 | 21 | 0 | 1 | 0 | 68 | 58 | 1 | 12 | 0 | 0 | 1 | 4 |
| *C.arvensis* | 0 | 1 | 0 | 0 | 0 | 0 | 0 | 0 | 0 | 0 | 0 | 0 | 0 |
| *C.pinetorum* | 0 | 4 | 0 | 0 | 0 | 3 | 0 | 0 | 0 | 0 | 0 | 0 | 0 |
| *C.thyrsoideum* | 1 | 157 | 7 | 6 | 28 | 4 | 1 | 28 | 104 | 4 | 0 | 0 | 23 |
| *C.watsoniana* | 0 | 2 | 0 | 0 | 0 | 0 | 2 | 0 | 0 | 0 | 0 | 0 | 0 |
| *L.mexicana* | 0 | 6 | 0 | 0 | 0 | 1 | 0 | 0 | 6 | 0 | 0 | 0 | 0 |
| *S.elegans* | 0 | 21 | 0 | 0 | 0 | 0 | 0 | 0 | 0 | 0 | 0 | 0 | 9 |
| *S.gesneriiflora* | 0 | 0 | 0 | 0 | 0 | 0 | 2 | 0 | 0 | 0 | 0 | 0 | 0 |
| *S.iodantha* | 1 | 1066 | 23 | 9 | 4 | 0 | 28 | 0 | 99 | 16 | 1 | 11 | 422 |
| *S.mexicana* | 0 | 6 | 0 | 0 | 0 | 0 | 0 | 0 | 0 | 0 | 0 | 0 | 0 |
