# Supplementary material for: Implications of dominance hierarchy on hummingbird-plant interactions in a temperate forest in Northwestern Mexico
Source: PeerJ. 2023 Oct 17;11:e16245. doi: 10.7717/peerj.16245 (PMC10588686; doi:10.7717/peerj.16245)
Supplement: Supplemental Information 4 [file peerj-11-16245-s004.docx]

Table S2. Hummingbird´s traits Principal Components Analysis (PCA).

- 1. PCA Variance percent

|  | Variance percent | Cumulative variance percent |
| --- | --- | --- |
| Dim 1 | 73.75 | 73.75 |
| Dim 2 | 22.15 | 95.90 |
| Dim 3 | 2.60 | 98.51 |
| Dim 4 | 1.48 | 100 |

2.2 Scores of the variables in the PCA. The highest contributions in each dimension are in bold.

|  | Dim 1 | Dim 2 | Dim 3 | Dim 4 |
| --- | --- | --- | --- | --- |
| Bill length | 0.897 | -0.369 | **0.235** | 0.048 |
| Weight | 0.961 | -0.137 | -0.201 | 0.128 |
| Bill curvature | 0.512 | **0.854** | 0.071 | 0.047 |
| Ds | **0.978** | 0.026 | -0.056 | **-0.195** |
