# Supplementary material for: Implications of dominance hierarchy on hummingbird-plant interactions in a temperate forest in Northwestern Mexico
Source: PeerJ. 2023 Oct 17;11:e16245. doi: 10.7717/peerj.16245 (PMC10588686; doi:10.7717/peerj.16245)
Supplement: Supplemental Information 5 [file peerj-11-16245-s005.docx]

Table S3. Hummingbird´s traits Principal Components Analysis (PCA).

3.1 PCA Variance percent

|  | Variance percent | Cumulative variance percent |
| --- | --- | --- |
| Dim 1 | 73.5 | 73.5 |
| Dim 2 | 22.5 | 96.0 |
| Dim 3 | 2.56 | 98.5 |
| Dim 4 | 1.44 | 100 |

3.2 Scores of the variables in the PCA. The highest contributions in each dimension are in bold.

|  | PC1 | PC2 | PC3 | PC4 |
| --- | --- | --- | --- | --- |
| Bill length | 0.525 | **0.384** | 0.680 | -0.335 |
| Weight | 0.559 | 0.144 | **-0.711** | -0.400 |
| Bill curvature | 0.288 | -0.911 | 0.175 | -0.235 |
| Perc | **-0.571** | 0.034 | 0.018 | **-0.819** |
