# Supplementary material for: Implications of dominance hierarchy on hummingbird-plant interactions in a temperate forest in Northwestern Mexico
Source: PeerJ. 2023 Oct 17;11:e16245. doi: 10.7717/peerj.16245 (PMC10588686; doi:10.7717/peerj.16245)
Supplement: Supplemental Information 6 [file peerj-11-16245-s006.docx]

Table S4. Plants traits Principal Components Analysis (PCA).

4.1 PCA Variance percent

|  | Variance percent | Cumulative variance percent |
| --- | --- | --- |
| Dim 1 | 52.133 | 52.133 |
| Dim 2 | 19.845 | 71.979 |
| Dim 3 | 16.735 | 88.418 |
| Dim 4 | 10.735 | 99.153 |
| Dim 5 | 0.840 | 99.994 |
| Dim 6 | 0.005 | 100 |

4.2 Scores of the variables in the PCA. The highest contributions in each dimension are in bold.

|  | Dim 1 | Dim 2 | Dim 3 | Dim 4 | Dim 5 | Dim 6 |
| --- | --- | --- | --- | --- | --- | --- |
| Volume | **-0.970** | -0.168 | 0.159 | -0.003 | 0.062 | **0.01** |
| Bix | 0.521 | -0.2484 | **0.602** | **0.549** | -0.028 | 0.001 |
| Calories | -0.925 | -0.279 | 0.216 | 0.089 | 0.098 | -0.01 |
| Length | -0.196 | **-0.739** | -0.593 | 0.238 | -0.064 | 0.0004 |
| Width | -0.933 | 0.199 | 0.235 | -0.043 | **-0.176** | -.0001 |
| Curvature | 0.382 | -0.659 | 0.377 | -0.524 | -0.028 | -9.32-05 |
