# Supplementary material for: Implications of dominance hierarchy on hummingbird-plant interactions in a temperate forest in Northwestern Mexico
Source: PeerJ. 2023 Oct 17;11:e16245. doi: 10.7717/peerj.16245 (PMC10588686; doi:10.7717/peerj.16245)
Supplement: Supplemental Information 7 [file peerj-11-16245-s007.docx]

Table S5. Bill morphology (curvature and length), Species-level within the interaction networks (d´ and degree), and Core-periphery for the 13 hummingbird species.

|  | Bill morphology | | Species-level network | |
| --- | --- | --- | --- | --- |
| Species | Curvature (°) | Length (mm) | Sqrt (d´) | Core-periphery |
| Berylline Hummingbird | 4.428 | 19.407 | 0.363 | periphery |
| Blue-throated Mountain-gem | 4.620 | 22.312 | 0.611 | periphery |
| Broad-billed Hummingbird | 3.816 | 20.147 | 0.160 | periphery |
| Broad-tailed Hummingbird | 3.809 | 18.217 | 0.185 | periphery |
| Bumblebee Hummingbird | 3.380 | 12.665 | 0 | periphery |
| Calliope Hummingbird | 3.330 | 15.645 | 0.127 | periphery |
| Costa’s Hummingbird | 3.989 | 17.285 | 0.145 | periphery |
| Mexican Violetear | 7.296 | 19.690 | 0.541 | periphery |
| Rivoli's Hummingbird | 3.539 | 27.444 | 0.821 | periphery |
| Ruby-throated Hummingbird | 3.748 | 17.512 | 0.104 | periphery |
| Rufous Hummingbird | 3.546 | 17.361 | 0.288 | Core |
| Violet-crowned Hummingbird | 3.729 | 22.530 | 0.623 | periphery |
| White-eared Hummingbird | 3.625 | 17.865 | 0.279 | Core |
